# Characteristics of above 65-year-olds with type 1 diabetes in the Finnish diabetic nephropathy study

**DOI:** 10.1007/s00592-025-02613-0

**Published:** 2025-11-18

**Authors:** Emilia M.C. Franzén, Marika I. Eriksson, Susanna Satuli-Autere, Anni Ylinen, Fanny Jansson Sigfrids, Jenna Nicklén, Hanna Öhman, Per-Henrik Groop, Lena M. Thorn, the FinnDiane Study Group

**Affiliations:** 1 Folkhälsan Research Center, Helsinki, Finland; 2 Research Program for Clinical and Molecular Metabolism, Faculty of Medicine, University of Helsinki, Helsinki, Finland; 3 Department of General Practice and Primary Health Care, University of Helsinki and Helsinki University Hospital, Helsinki, Finland; 4 Department of Nephrology, University of Helsinki and Helsinki University Hospital, Helsinki, Finland; 5 Department of Geriatrics, University of Helsinki and Helsinki University Hospital, Helsinki, Finland; 6 Department of Diabetes, Central Clinical School, Monash University, Melbourne, VIC Australia; 7 Baker Heart and Diabetes Institute, Melbourne, VIC Australia

**Keywords:** Ageing, Cardiometabolic risk factors, Diabetes complications, Diabetes mellitus, type 1

## Abstract

**Aims:**

Ageing in people with type 1 diabetes is identified as a research gap. Therefore, the aim of our study is to characterize above 65-year-olds with type 1 diabetes, and to identify potential protective factors or factors related to increased risk of mortality in this age group.

**Methods:**

This observational study includes 864 participants aged 55 years or older with type 1 diabetes (age at onset below 40) from the Finnish Diabetic Nephropathy Study, grouped according to age into three categories: 55–60, 60–65, and > 65 years old. Multivariable logistic regression analysis was used to identify factors independently associated with age above 65. Cox regression analysis was conducted to assess how these factors impact survival.

**Results:**

Factors that were independently associated with age above 65 years included: higher diabetes onset age, higher pulse pressure, lower mean arterial pressure, absence of current smoking and diabetic kidney disease, history of severe diabetic retinopathy and cardiovascular events, lower daily insulin dose, lower HbA_1c_, and lowerApoB-100 concentrations. Of these factors, the ones associated with mortality in above 65-year-olds during follow-up were presence of diabetic kidney disease, higher HbA_1c_, and history of cardiovascular events.

**Conclusion:**

Above 65-year-olds were characterized by both factors generally related to positive and negative health outcomes. Additionally, different factors were found to be associated with reaching older age and with survival beyond the age of 65.

**Supplementary Information:**

The online version contains supplementary material available at 10.1007/s00592-025-02613-0.

## Introduction

Ageing in people with type 1 diabetes is becoming a common phenomenon due to improved treatment of diabetes and its long-term complications. People with type 1 diabetes live longer than before, but their life expectancy is still shorter than that of the general population [1], mainly due to acute and especially chronic complications of diabetes [2].

Ageing is often associated with various challenges, such as multimorbidity, polypharmacy, cognition, and frailty, and these phenomena are accentuated in people with diabetes [3, 4]. However, data specific to type 1 diabetes in this context remain limited [5]. It is well-known that the cumulative incidence of long-term micro- and macrovascular complications related to diabetes increase with age, and especially with longer duration of type 1 diabetes [6–9]. In type 1 diabetes, the aging-related increase in systolic blood pressure (SBP) and the decrease in diastolic blood pressure (DBP) occurs 15–20 years earlier compared to the general population, resulting in higher pulse pressure (PP) [10], an indicator of arterial stiffening, which is a strong predictor of cardiovascular disease [11]. There are studies indicating that the risk of severe hypoglycemia is progressively increasing with age and that the HbA_1c_ concentrations are lower in older individuals with type 1 diabetes [12].

Studies assessing the impact of long duration of type 1 diabetes, such as the UK Golden Years Cohort and the Joslin Medalist Study of individuals with more than 50 years of diabetes, have identified protective factors related to long duration, such as elevated HDL cholesterol, healthy lifestyle, and absence of kidney disease [13, 14]. These studies indicate that those who endure a long duration of diabetes, can at least partially be considered survivors, since they are characterized by factors related to positive health outcomes, while those with high complication burden face a shorter lifespan. Long diabetes duration does not, however, equal old age, since the diabetes onset age can vary. People with older age can have a shorter duration of type 1 diabetes, and people with even 50 years of diabetes duration might still be middle-aged.

In clinical practice, older adults with type 1 diabetes are a heterogenous group, and we lack data to support their treatment. In line with this, the impact of ageing in type 1 diabetes has been identified as a research gap [5]. The aim of our study is, thus, to assess characteristics related to ageing, by comparing people aged above 65 years living with type 1 diabetes to those 55–60 years and those aged 60–65 years, in a comprehensively characterized cohort of individuals with type 1 diabetes in Finland. In addition, we aim to assess how these ageing-related factors contribute to survival in above 65-year-olds with type 1 diabetes.

## Methods

### Participants

All participants are part of the Finnish Diabetic Nephropathy (FinnDiane) Study, an ongoing, nationwide multicenter study with the aim to uncover genetic and environmental risk factors for long-term complications of type 1 diabetes. The FinnDiane Study was initiated in 1997, and 5,473 people with type 1 diabetes have been enrolled across any of the 77 study centers around Finland (Supplemental Table 1). The study has been described in detail previously [15].

For this cross-sectional study, we included all 864 participants with type 1 diabetes being older than 55 years at any of their baseline or follow-up visits ranging from 1997 to 2023 (median 2011, interquartile range 2005–2016). Of them, 536 (62%) had attended one or more follow-up visits and we used the data from the most recent study visit. To verify a correct diagnosis of type 1 diabetes, the individuals had to been diagnosed under the age of 40 and had started insulin treatment within a year after the diagnosis. The participants were categorized into three groups according to age: 55–60, 60–65, and > 65 years old. None of the participants was exactly 60.

### FinnDiane study visit

The FinnDiane study visit included a comprehensive clinical evaluation. Information concerning diabetes history, diabetic complications, and medication was recorded by the attending physician or research nurse, who verified the information from the medical records. The anthropometric data included measurements of body weight, height, and waist circumference. Office blood pressure (BP) was measured twice, and the mean value was used for analysis. Waistline was measured midway between the lowest ribs and the iliac crest. PP was calculated as SBP minus DBP and mean arterial pressure (MAP) as DBP plus 1/3 (SBP-DBP) in mmHg.

Blood tests were taken for analysis of HbA_1c_, lipids and lipoproteins, creatinine, cystatin C, and high sensitivity C-reactive protein (hs-CRP). LDL cholesterol was calculated according to the Sampson model [16]. The estimated glomerular filtration rate (eGFR) was estimated with the Chronic Kidney Disease Epidemiology Collaboration (CKD-EPI) equation from 2009 [17]. Moderately increased albuminuria was defined based on two out of three consecutive urine collections as urinary albumin excretion rate > 20 and ≤ 200 µg/min or > 30 and ≤ 300 mg/24 h or urinary albumin-to-creatinine ratio > 3 and ≤ 30 mg/mmol and diabetic kidney disease (DKD) as severely increased albuminuria (> 200 µg/min, > 300 mg/24 h, or > 30 mg/mmol) or kidney replacement therapy, including ongoing dialysis treatment or having received a previous kidney transplant. Severe diabetic retinopathy was defined as a history of retinal photocoagulation. History of cardiovascular events includes acute myocardial infarction, coronary revascularization, stroke, lower-limb amputation, or peripheral artery revascularization. Information on smoking was self-reported and current smoking was defined as smoking at least one cigarette per day for at least one year. Depression was defined based on self-reported data or by current use of antidepressants. Education was determined from self-reported data on the number of years of education after primary and lower secondary education (comprehensive school). Occupation was categorized based on self-reported data on occupation and educational attainment to blue collar workers vs. all other categories.

### Follow-up data on mortality

Follow-up data on mortality were available for all participants and were retrieved from the National Cause of Death Registry, Statistics Finland. Follow-up time was calculated from the FinnDiane study visit until death from any cause or end of follow-up on December 31st, 2020.

### Statistical analysis

To compare the characteristics of those above 65-year-olds to those 55–60 years old and 60–65 years old, normally distributed continuous variables were analyzed with one-way ANOVA and the results are presented as means with standard deviations, non-normally distributed variables were tested with the Kruskal Wallis test and the results are presented as medians with interquartile ranges, and categorical variables were analyzed with the χ^2^ test with the results presented as *n* with percentages. Multivariable logistic regression analyses with a manual forward stepwise approach, adjusted for sex, were used to determine factors independently associated with age above 65 years. Age and diabetes duration were not included in the models due to strong correlation with the dependent variable. The results are presented as odds ratios (OR) with 95% confidence intervals (CI). Survival is plotted with Kaplan-Meier curves with between-group comparisons analyzed with the log rank test. Univariable and multivariable Cox regression analyses were conducted to assess how factors independently associated with age above 65 years in a cross-sectional setting further impact subsequent survival. These results are presented with hazard ratios (HR) with 95% CIs. A P-value < 0.05 was considered statistically significant. All analyses were performed using IBM SPSS statistics version 28.

## Results

Of the 864 participants, 401 (46.4%) were between 55 and 60, 239 (27.7%) between 60 and 65, and 224 (25.9%) above 65 years. Clinical characteristics of the participants according to these three age groups are presented in Table 1. The groups were similar regarding sex-distribution, body mass index (BMI), and waist-height ratio, while age at diabetes onset increased with age. SBP also increased with age, while DBP decreased. Consequently, MAP decreased with age, and PP increased. Total cholesterol and non-HDL cholesterol decreased with age. Both the daily insulin dose and HbA_1c_ decreased with age, while hs-CRP did not differ between the groups. DKD was less prevalent with increasing age, while the other complications (severe diabetic retinopathy and cardiovascular disease) were more prevalent. Current smoking was less prevalent with older age. No differences between groups were observed for the presence of depression, education, or occupational category. Pension or disability pension was the most prevalent in above 65-year-olds but was also high in the other age groups.

**Table 1:** Characteristics of the study cohort based on age groups: 55–60, 60–65, and above 65 years of age

	*N*	Ages 55–60 *n* = 401	Ages 60–65 *n* = 239	Ages > 65 *n* = 224	*P*-value
Men, n (%)	864	210 (52.4)	126 (52.7)	132 (58.9)	0.139*
Age, years	864	57.2 (56.1–58.6)	61.8 (61.0–63.5)	68.8 (66.7–72.4)	< 0.001
Diabetes duration, years	864	40.3 (30.1–46.7)	43.2 (36.1–49.3)	47.8 (39.9–53.7)	< 0.001
Diabetes onset age, years	864	17.1 (10.9–27.4)	19.2 (12.8–25.8)	22.7 (15.9–29.4)	< 0.001
Waist-height ratio	834	0.54 ± 0.07	0.54 ± 0.07	0.54 ± 0.07	0.523
BMI, kg/m^2^	848	26.3 ± 4.2	26.3 ± 4.1	25.7 ± 3.8	0.148
Systolic blood pressure, mmHg	841	147 ± 19	150 ± 20	150 ± 21	0.031
Diastolic blood pressure, mmHg	841	78 ± 9	75 ± 10	71 ± 10	< 0.001
Mean arterial pressure, mmHg	841	101 ± 11	100 ± 11	98 ± 12	0.004
Pulse pressure, mmHg	841	69 ± 16	75 ± 18	78 ± 18	< 0.001
Heart rate, bpm	630	71 ± 11	69 ± 11	68 ± 11	0.009
Antihypertensive medication, n (%)	854	284 (71.5)	187 (79.6)	179 (80.6)	0.006*
RAAS inhibitor, n (%)	849	222 (56.3)	142 (60.9)	142 (64.0)	0.057*
Total cholesterol, mmol/l	855	4.74 ± 0.97	4.64 ± 1.00	4.52 ± 0.91	0.018
LDL cholesterol, mmol/l	855	2.5 (2.0–3.2)	2.4 (1.9–3.1)	2.3 (1.7–3.0)	0.002
HDL cholesterol, mmol/l	855	1.5 (1.2–1.9)	1.6 (1.2–1.9)	1.6 (1.3–1.9)	0.153
Triglycerides, mmol/l	855	1.05 (0.80–1.46)	1.05 (0.80–1.44)	1.44 (0.76–1.34)	0.199
Serum ApoA1, mg/dl	822	145 (127–169)	143 (125–160)	140 (121–158)	0.024
Serum ApoB-100, mg/dl	822	73 (61–88)	71 (59–88)	66 (54–81)	< 0.001
Non-HDL cholesterol, mmol/l	840	3.03 (2.49–3.71)	2.92 (2.43–3.60)	2.75 (2.21–3.44)	< 0.001
Lipid-lowering medication, n (%)	852	209 (52.6)	133 (57.1)	132 (59.5)	0.139 *
Insulin dose, IU/kg	850	0.56 ± 0.28	0.54 ± 0.25	0.48 ± 0.24	< 0.001
HbA_1c_ (mmol/mol)	833	66 ± 13	64 ± 13	61 ± 12	< 0.001
HbA_1c_ (%)	833	8.2 ± 1.1	8.0 ± 1.2	7.7 ± 1.1	< 0.001
hs-CRP, mg/l	843	1.45 (0.73–3.04)	1.43 (0.70–3.14)	1.24 (0.65–2.84)	0.375
Creatinine, µmol/l	856	81 (65–109)	81 (68–117)	83 (71–104)	0.510
eGFR, ml/min/1.73m^2^	856	82.8 (61.7–97.9)	79.7 (56.1–93.1)	74.5 (58.7–88.4)	< 0.001
eGFR < 60 ml/min/1.73 m^2^, n (%)	856	93 (23.5)	66 (27.7)	59 (26.5)	0.466
Cystatin C, mg/l	391	0.99 (0.84–1.39)	1.05 (0.87–1.47)	1.15 (0.97–1.52)	0.010
Moderate albuminuria, n (%)	834	57 (14.6)	37 (16.2)	44 (20.5)	0.069*
Diabetic kidney disease, n (%)	834	123 (31.5)	74 (32.5)	42 (19.5)	0.003
Kidney replacement therapy, n (%)	834	52 (13.3)	31 (13.6)	12 (5.6)	0.008
Severe diabetic retinopathy, n (%)	845	205 (52.3)	139 (59.1)	134 (61.5)	0.021*
Cardiovascular event, n (%)	854	123 (31.0)	85 (36.2)	96 (43.2)	0.002*
Current smoking, n (%)	826	49 (12.9)	17 (7.5)	9 (4.1)	< 0.001*
History of smoking, n (%)	826	185 (48.7)	93 (41.2)	89 (40.5)	0.036*
Depression, n (%)	787	38 (10.6)	20 (9.3)	19 (8.9)	0.497*
Years of education after comprehensive school, years	506	5 (2–7)	5 (3–7)	4 (2–8)	0.731
Blue-collar worker, n (%)	681	190 (59.7)	106 (57.3)	106 (59.6)	0.853
Pension or disability pension, n (%)	717	156 (46.6)	137 (71.0)	177 (93.7)	< 0.001*

Data are presented as mean ± standard deviation, median (interquartile range), or n (%). BMI: body mass index; RAAS: renin-angiotensin-aldosterone system; LDL: low density lipoprotein; HDL: high density lipoprotein; hs-CRP: high sensitivity C-reactive protein; eGFR: estimated glomerular filtration rate

*P-value for trend

We further assessed which factors were independently associated with age above 65 years using multivariable logistic regression analysis (Table 2). The factors identified were higher PP, higher diabetes onset age, lower MAP, absence of DKD, non-smoking status, a history of severe retinopathy, lower daily insulin dose, lower HbA_1c_, lower ApoB-100 concentration, and history of cardiovascular events. Of note, ApoB-100 was also independently and negatively associated with above 65 years of age after adjustment for lipid-lowering medication (data not shown).

**Table 2:** Factors independently associated with above 65 years of age

	OR	95% CI	*P*-value
Pulse pressure, per 1 mmHg	1.05	1.04–1.07	< 0.001
Diabetes onset age, per 1 year	1.09	1.07–1.12	< 0.001
Mean arterial pressure, per 1 mmHg	0.94	0.92–0.96	< 0.001
Diabetic kidney disease, yes vs. no	0.35	0.21–0.58	< 0.001
Current smoking, yes vs. no	0.23	0.10–0.53	< 0.001
Severe diabetic retinopathy, yes vs. no	2.00	1.30–3.07	0.002
Insulin dose, per 1 IU/kg	0.30	0.13–0.69	0.005
HbA_1c_, per 1 mmol/mol	0.98	0.96–0.99	0.009
ApoB-100, per 1 mg/dl	0.99	0.98–0.997	0.014
Cardiovascular event, yes vs. no	1.60	1.04–2.45	0.031

Multivariable sex-adjusted logistic regression analysis, with data presented as the odds ratio (OR) with 95% confidence interval (CI)

During a median follow-up of 7.12 (3.72–12.56) years, 309 (35.9%) died, and of these deaths 226 (73,1) were due to cardiovascular or diabetes-related causes. The frequency of deaths increased with baseline age: 139 deaths (32.6%) in participants 55–60 years, 86 deaths (36.1%) in those aged 60–65 years, and 93 deaths (41.7%) in those above 65 years; *P* = 0.024 for trend. Follow-up time decreased with increasing age: 55–60 years: 8.1 years (4.1–14.2), 60–65 years: 6.9 years (3.7–12.2), and above 65 years: 5.0 years (3.2–8.6), *P* < 0.001. In Kaplan-Meier survival analysis, survival decreased with increasing age, *P* < 0.05 for pair-wise comparisons between age-groups (Supplemental Fig. 1).

To test how the factors independently associated with age above 65 contribute to their survival in a longitudinal setting, we performed Cox regression analyses (Table 3). In the multivariable Cox regression model, DKD, HbA_1c_, and a history of a cardiovascular event were positively and independently associated with all-cause mortality.

**Table 3:** Univariable and multivariable Cox regression analyses for all-cause mortality in the above 65-year-olds

	Univariable analyses		Multivariable analysis	
	HR (95% CI)	P-value	HR (95% CI)	P-value
Pulse pressure, per 1 mmHg	1.00 (0.99–1.01)	0.849	-	-
Diabetes onset age, per 1 year	1.02 (0.99–1.04)	0.210	-	-
Mean arterial pressure, per 1 mmHg	1.00 (0.98–1.02)	0.901	-	-
Diabetic kidney disease, yes vs. no	4.10 (2.56–6.57)	< 0.001	3.32 (1.99–5.53)	< 0.001
Current smoking, yes vs. no	0.83 (0.26–2.64)	0.749	-	-
Severe diabetic retinopathy, yes vs. no	2.08 (1.30–3.31)	0.002	1.28 (0.76–2.16)	0.351
Insulin dose, per 1 IU/kg	1.26 (0.47–3.32)	0.650	-	-
HbA_1c_, per 1 mmol/mol	1.02 (1.00–1.04)	0.019	1.03 (1.01–1.05)	0.007
ApoB-100, per 1 mg/dl	1.01 (0.99–1.01)	0.663	-	-
Cardiovascular event, yes vs. no	1.88 (1.24–2.85)	0.003	1.67 (1.06–2.63)	0.028

Data are presented as hazard ratios (HR) with 95% confidence interval (CI). All analyses are adjusted for sex

## Discussion

In this study of comprehensively characterized above 55-year-olds with type 1 diabetes, we observed that many characteristics showed either a decreasing or increasing trend with increasing age. Factors with an independent association with age above 65 were higher diabetes onset age, higher PP, lower MAP, absence of current smoking and DKD, history of severe diabetic retinopathy and cardiovascular events, lower daily insulin dose, lower HbA_1c_, and lower ApoB-100 concentration. Of these factors, the ones associated with mortality in the above 65-year-olds during follow-up were presence of DKD, higher HbA_1c_, and a history of cardiovascular events, indicating that different factors relate to reaching older age and to further survival in above 65-year-olds.

Of the factors independently associated with age above 65, some were confirmatory and in line with previous studies and some were novel. One of the novel findings was the higher diabetes onset age in the above 65-year-olds. This finding is partly expected, since higher diabetes onset age translates into shorter diabetes duration, and lower likelihood of severe complications related to longstanding hyperglycemia impacting survival. In line with this, individuals with diabetes onset in childhood are at exceptionally high risk of dying early [18, 19].

Our result of a higher PP with increasing age is in line with, and further adds to, the findings from younger individuals with type 1 diabetes reporting a linear increase in PP after 40 years of age [10]. Here, we also show that this increase in PP continues up to above 65-year-olds. The ageing-related increase in SBP and decrease in DBP may have a diluting impact on MAP. In the general population, the increase in MAP levels off at 50 to 60 years of age and decreases after the age of 70 to 80 [20]. Given the earlier rise in PP in type 1 diabetes [10], this ageing-related decrease in MAP could occur earlier in individuals with type 1 diabetes, explaining our findings of lower MAP in above 65-year-olds. Another explanation could be a survival bias related to better BP control, but there were no marked differences in SBP or proportion on antihypertensive medication between those above and below 65 years. Of note, and most of the study cohort was on antihypertensive medication, however, with unmet treatment targets especially for systolic blood pressure, reflecting either suboptimal treatment or resistant hypertension.

The lower likelihood of smoking, only 4.1%, in those above 65-year-olds represents a healthier lifestyle in those reaching an older age. The Swedish Diabetes Registry has also reported a declining trend in smoking with longer diabetes duration [21]. In our cohort, the difference in history of smoking was not as marked as for the current smoking, indicating that a higher proportion of those above 65-year-old managed to quit smoking, which could have translated into better survival, given the strong association between smoking and mortality [22]. Interestingly, psychosocial factors, such as depression, education, or occupation did not differ between the age groups.

 DKD was also clearly less common in the above 65-year-olds, suggesting that the high mortality rates related to DKD [2] translates into increased likelihood of reaching older age in those without kidney disease. In line with this, DKD showed a strong association with mortality also in the present study. There are, however, studies reporting the opposite that the DKD prevalence increases with age [23], and that moderate and severe albuminuria are more common in those above 60 years [12]. In our study, the prevalence of moderate albuminuria did not differ by age-groups. Beyond albuminuria, we also compared different measures of kidney function between the groups, but the measures such as Cystatin C or creatinine-based eGFR < 60 ml/min/1.73^2^ were not independently associated with older age. Shifting the perspective from age to diabetes duration, it is noteworthy that also these studies report conflicting results, with some demonstrating a high cumulative incidence of severe DKD [24], while others have found a low prevalence of complications [14] with more than 50 years of type 1 diabetes. In other words, the observed discrepancies could reflect differences in cohort recruitment or cohort characteristics. It is also possible that treatment modalities available at different time periods affect the results. It is known that the incidence decline in diabetic kidney disease has plateaued after an initial decrease [25], probably due to the introduction of renin–angiotensin–aldosterone system inhibitors. This also highlights the urgent need for additional kidney and cardioprotective medication, such as the SGLT2 inhibitors, GLP-1 receptor agonists, and the non-steroidal mineralocorticoid receptor antagonists finerenone [26], to be available also for individuals with type 1 diabetes.

 For the other diabetic complications that we assessed, i.e. diabetic retinopathy and cardiovascular events, we observed an increasing prevalence with increasing age, also reported by others [12, 27]. In our above 65-year-olds, 61.5% had severe diabetic retinopathy, which is in the same magnitude as reported in the Joslin Medalist Study with > 50 years of diabetes duration (57.4%). For cardiovascular events, the prevalence in above 65-year-olds was as high as 43.2%, in line with results from the UK general practice database (39.5%) [27], but higher than that reported from German > 60-year-olds [27]. Taken together, the burden of cardiovascular disease in older adults with type 1 diabetes is high, as also noted by population-based studies reporting a several-fold increase in cardiovascular disease incidence related to type 1 diabetes [28]. Of note, our participants aged 55–65 also showed a rather high prevalence of both micro- and macrovascular complications, prodisease incidence related to typebably explaining the high proportion on disability pension these age groups.

 As expected, the above 65-year-olds had lower HbA_1c_ concentrations than the younger age groups, as reported previously [12]. In the current study, the younger age-groups 55–65 did not meet the treatment targets regarding mean HbA_1c_. The lower HbA_1c_ in the above 65-year-olds can be an indication of survival bias, also supported by the higher HbA_1c_ associated with increased risk of all-cause mortality in the above 65-year-olds. A lower HbA_1c_ by older age could also result from changes in body composition, physical activity, nutrition, and insulin sensitivity with older age, but these alterations are still poorly understood in type 1 diabetes [5].

 Interestingly, the insulin dose per body weight was also lower for the above 65-year-olds, which could reflect better insulin sensitivity. Insulin resistance is a known risk factor for micro- and macrovascular complications in individuals with type 1 diabetes [29] potentially impacting survival of our cohort. However, chronic inflammation, assessed by hs-CRP, also known to associate with insulin resistance [10] did not differ between the age groups, but it is noteworthy that the LDL cholesterol concentrations were suboptimal according to current guidelines [30].

Of the lipids and apolipoproteins, only ApoB-100 was independently and negatively associated with above 65 years of age, indicating a slightly more favorable lipid profile with increasing age, as lower ApoB-100 reflects lower number of circulating atherogenic lipoprotein particles and is, thus, associated with reduced cardiovascular risk [31]. Of note, use of lipid-lowering medication did not differ between the age groups.

 Strengths of this study include the comprehensively characterized nationwide cohort of people with type 1 diabetes and a long follow-up period for mortality. However, with the observational nature, we cannot draw any definite conclusions regarding the protective vs. risk-increasing role of the identified factors but can only speculate on their role. Furthermore, we could not account for some important factors that we did not have complete data on, such as history of hypoglycemia, physical activity, diet, alcohol consumption, and functional ability.

Further studies are needed to evaluate how ageing influences people with type 1 diabetes, especially regarding the potential survival bias vs. the long-term harmful effects of longstanding dysglycemia related to type 1 diabetes.

## Supplementary Information

Below is the link to the electronic supplementary material.

Supplementary Material 1

## Data Availability

Individual-level data for the study participants are not publicly available due to the local legislation and the written consents by the participant at the time of data collection. Data that support the findings are available from the corresponding author/lead investigator upon reasonable request.

## References

[1] Arffman M, Hakkarainen P, Keskimäki I, Oksanen T, Sund R (2023) Long-term and recent trends in survival and life expectancy for people with type 1 diabetes in Finland. Diabetes Res Clin Pract 198:110580. 10.1016/j.diabres.2023.110580 36804193 10.1016/j.diabres.2023.110580

[2] Groop PH, Thomas MC, Moran JL, Waden J, Thorn LM, Makinen VP et al (2009) The presence and severity of chronic kidney disease predicts all-cause mortality in type 1 diabetes. Diabetes;58(7):1651-8. 10.2337/db08-1543 10.2337/db08-1543 PMC2699848 19401416

[3] Sinclair AJ, Abdelhafiz AH (2022) Multimorbidity, frailty and diabetes in older People-Identifying interrelationships and outcomes. Personalized Med 12(11):1911. 10.3390/jpm12111911 10.3390/jpm12111911 PMC9695437 36422087

[4] Remelli F, Ceresini MG, Trevisan C, Noale M, Volpato S (2022) Prevalence and impact of polypharmacy in older patients with type 2 diabetes. Aging Clin Exp Res 34(9):1969–1983. 10.1007/s40520-022-02165-1 35723858 10.1007/s40520-022-02165-1 PMC9464133

[5] Munshi MN, Meneilly S, Rodríguez-Mañas G, Conlin L, Cukierman-Yaffe PR, Forbes T A, et al (2020) Diabetes in ageing: pathways for developing the evidence base for clinical guidance. Lancet Diabetes Endocrinol 8(10):855–867. 10.1016/S2213-8587(20)30230-8 32946822 10.1016/S2213-8587(20)30230-8 PMC8223534

[6] Young MJ, Boulton AJ, MacLeod AF, Williams DR, Sonksen PH (1993) A multicentre study of the prevalence of diabetic peripheral neuropathy in the united Kingdom hospital clinic population. Diabetologia 36(2):150–154. 10.1007/BF00400697 8458529 10.1007/BF00400697

[7] Henricsson M, Nilsson A, Groop L, Heijl A, Janzon L (1996) Prevalence of diabetic retinopathy in relation to age at onset of the diabetes, treatment, duration and glycemic control. Acta Ophthalmol Scand 74(6):523–527. 10.1111/j.1600-0420.1996.tb00727.x 9017034 10.1111/j.1600-0420.1996.tb00727.x

[8] Raile K, Galler A, Hofer S, Herbst A, Dunstheimer D, Busch P, Holl RW (2007) Diabetic nephropathy in 27,805 children, adolescents, and adults with type 1 diabetes: effect of diabetes duration, A1C, hypertension, dyslipidemia, diabetes onset, and sex. Diabetes Care 30(10):2523–2528. 10.2337/dc07-0282 17630266 10.2337/dc07-0282

[9] Harjutsalo V, Barlovic DP, Gordin D, Forsblom C, King G, Groop PH, FinnDiane Study G (2021) Presence and determinants of cardiovascular disease and mortality in individuals with type 1 diabetes of long duration: the FinnDiane 50 years of diabetes study. Diabetes Care 44(8):1885–1893. 10.2337/dc20-2816 34162664 10.2337/dc20-2816

[10] Ronnback M, Fagerudd J, Forsblom C, Pettersson-Fernholm K, Reunanen A, Groop P (2004) Altered age-related blood pressure pattern in type 1 diabetes. Circulation 110(9):1076–1082. 10.1161/01.CIR.0000139903.29522.8D 15326070 10.1161/01.CIR.0000139903.29522.8D

[11] Franklin SS, Khan SA, Wong ND, Larson ND, Levy D (1999) Is pulse pressure useful in predicting risk for coronary heart disease? The Framingham heart study. Circulation 100(4):354–360. 10.1161/01.cir.100.4.354 10421594 10.1161/01.cir.100.4.354

[12] Schutt M, Fach EM, Seufert J, Kerner W, Lang W, Zeyfang A et al (2012) Multiple complications and frequent severe hypoglycaemia in ‘elderly’ and ‘old’ patients with type 1 diabetes. Diabet Med 29(8):176–179. 10.1111/j.1464-5491.2012.03681.x 22506989 10.1111/j.1464-5491.2012.03681.x

[13] Bain SC, Gill GV, Dyer PH, Jones AF, Murphy M, Jones KE et al (2003) Characteristics of type 1 diabetes of over 50 years duration (the golden years Cohort). Diabet Med 20(10):808–811. 10.1046/j.1464-5491.2003.01029.x 14510860 10.1046/j.1464-5491.2003.01029.x

[14] Sun J, Keenan H, Cavallerano J, Asztalos B, Schaefer E, Sell D et al (2011) Protection from retinopathy and other complications in patients with type 1 diabetes of extreme duration: the Joslin 50-year medalist study. Diabetes Care 34(4):968–974. 10.2337/dc10-1675 21447665 10.2337/dc10-1675 PMC3064059

[15] Hägg S, Thorn L, Putaala J, Liebkind R, Harjutsalo V, Forsblom C et al (2013) Incidence of stroke according to presence of diabetic nephropathy and severe diabetic retinopathy in patients with type 1 diabetes. Diabetes Care 36(12):4140–4146. 10.2337/dc13-0669 24101700 10.2337/dc13-0669 PMC3836162

[16] Sampson M, Ling C, Sun Q, Harb R, Ashmaig M, Warnick R et al (2020) A new equation for calculation of Low-Density lipoprotein cholesterol in patients with normolipidemia and/or hypertriglyceridemia. JAMA Cardiol 5(5):540–548. 10.1001/jamacardio.2020.0013 32101259 10.1001/jamacardio.2020.0013 PMC7240357

[17] Levey AS, Stevens LA, Schmid CH, Zhang YL, Castro AF, Feldman HI et al (2009) A new equation to estimate glomerular filtration rate. Ann Intern Med 150(9):604–612. 10.7326/0003-4819-150-9-200905050-00006 19414839 10.7326/0003-4819-150-9-200905050-00006 PMC2763564

[18] Harjutsalo V, Maric-Bilkan C, Forsblom C, Groop P-H (2014) Impact of sex and age at onset of diabetes on mortality from ischemic heart disease in patients with type 1 diabetes. Diabetes Care 37(1):144–148. 10.2337/dc13-0377 24062319 10.2337/dc13-0377

[19] Rawshani A, Sattar N, Franzén S, Rawshani A, Hattersley A, Svensson A et al (2018) Excess mortality and cardiovascular disease in young adults with type 1 diabetes in relation to age at onset: a nationwide, register-based cohort study. Lancet 11(392):477–486. 10.1016/S0140-6736(18)31506-X 10.1016/S0140-6736(18)31506-X PMC6828554 30129464

[20] Franklin SS, Gustin W, Wong ND, Larson MG, Weber MA, Kannel WB, Levy D (1997) Hemodynamic patterns of age-related changes in blood pressure. Framingham Heart Study Circulation 96(1):308–315. 10.1161/01.cir.96.1.308 9236450 10.1161/01.cir.96.1.308

[21] Adamsson Eryd S, Svensson A, Franzén S, Eliasson B, Nilsson P, Gudbjörnsdottir S (2017) Risk of future microvascular and macrovascular disease in people with type 1 diabetes of very long duration: a National study with 10-year follow-up. Diabet Med 34(3):411–418. 10.1111/dme.13266 27647178 10.1111/dme.13266 PMC5516128

[22] Pan A, Wang Y, Talaei M, Hu F (2015) Relation of Smoking With Total Mortality and Cardiovascular Events Among Patients With Diabetes Mellitus: A Meta-Analysis and Systematic Review. Circulation;132(19):1795 – 804. 10.1161/CIRCULATIONAHA.115.017926 26311724 10.1161/CIRCULATIONAHA.115.017926 PMC4643392

[23] Pacilli A, Viazzi F, Fioretto P, Giorda C, Ceriello A, Genovese S et al (2016) Epidemiology of diabetic kidney disease in adult patients with type 1 diabetes in italy: the AMD-Annals initiative. Diabetes Metabolism 33(14). 10.1002/dmrr.2873 10.1002/dmrr.2873 27935651

[24] Costacou T, Orchard T (2018) Cumulative kidney complication risk by 50 years of type 1 diabetes: the effects of Sex, Age, and calendar year at onset. Diabetes Care 41(3):426–433. 10.2337/dc17-1118 28931542 10.2337/dc17-1118 PMC5829956

[25] Jansson Sigfrids F, Groop P-H, V H (2022) Incidence rate patterns, cumulative incidence, and time trends for moderate and severe albuminuria in individuals diagnosed with type 1 diabetes aged 0–14 years: a population-based retrospective cohort study. Lancet Diabetes Endocrinol 10(7):489–498. 10.1016/S2213-8587(22)00099-7 35489369 10.1016/S2213-8587(22)00099-7

[26] Rossing P, Caramori M, Chan J, Heerspink H, Hurst C, Khunti K et al (2022) KDIGO 2022 clinical practice guideline for diabetes management in chronic kidney disease. Kidney Int 102(5):1–127. 10.1016/j.kint.2022.06.008 10.1016/j.kint.2022.06.008 36272764

[27] Chapman M, Crockett S, Purvis T, Anderson M, Whittaker P, Bhattacharjee R et al (2013) Macrovascular disease in the elderly with type 1 diabetes. Diabetes Metab 4(9):426–433. 10.4172/2155-6156.1000299

[28] Harjutsalo VPBD, Groop P-H (2021) Long-term population-based trends in the incidence of cardiovascular disease in individuals with type 1 diabetes from finland: a retrospective, nationwide, cohort study. Lancet Diabetes Endocrinol 9(9):575–585. 10.1016/S2213-8587(21)00172-8 34303414 10.1016/S2213-8587(21)00172-8

[29] Bjornstad P, Snell-Bergeon J, Nadeau K, Maahs D (2015) Insulin sensitivity and complications in type 1 diabetes: new insights. World J Diabetes 6(1):8–16. 10.4239/wjd.v6.i1.8 10.4239/wjd.v6.i1.8 PMC4317319 25685274

[30] ADAPP Committee (2025) American diabetes association professional practice Committee; 10. Cardiovascular disease and risk management: standards of care in diabetes—2025. Diabetes Care 48(Supplement1):207–238. 10.2337/dc25-S010 10.2337/dc25-S010 PMC11635050 39651970

[31] Robinson JG, Wang S, Jacobson TA (2012) Meta-analysis of comparison of effectiveness of lowering apolipoprotein B versus low-density lipoprotein cholesterol and nonhigh-density lipoprotein cholesterol for cardiovascular risk reduction in randomized trials. Preventive cardiology 110(10):1468-76. 10.1016/j.amjcard.2012.07.007 10.1016/j.amjcard.2012.07.007 22906895

