# Supplementary material for: Characteristics of above 65-year-olds with type 1 diabetes in the Finnish diabetic nephropathy study
Source: Acta Diabetol. 2025 Nov 18;63(2):267–75. doi: 10.1007/s00592-025-02613-0 (PMC12956997; doi:10.1007/s00592-025-02613-0)

**Supplemental material**

**Acta Diabetologica**

**Characteristics of above 65-year-olds with type 1 diabetes in the Finnish Diabetic Nephropathy Study**

Emilia Franzén^1,2,3^, Marika I Eriksson^1,2,3,4^, Susanna Satuli-Autere^1,2,3^, Anni Ylinen^1,2,3,4^, Fanny Jansson Sigfrids^1,2,4^, Jenna Nicklén^1,2,3^, Hanna Öhman, Per-Henrik Groop^1,2,4,5,6^, Lena M Thorn^1,2,3, *^, on behalf of the FinnDiane Study Group

*^1^Folkhälsan Research Center, Helsinki, Finland*

*^2^Research Program for Clinical and Molecular Metabolism, Faculty of Medicine, University of Helsinki, Finland*

*^3^Department of General Practice and Primary Health Care, University of Helsinki and Helsinki University Hospital, Finland*

*^4^Department of Nephrology, University of Helsinki and Helsinki University Hospital, Helsinki, Finland*

*^5^Department of Geriatrics, University of Helsinki and Helsinki University Hospital, Helsinki, Finland*

*^6^Department of Diabetes, Central Clinical School, Monash University, Melbourne, VIC, Australia*

*^7^Baker Heart and Diabetes Institute, Melbourne, VIC, Australia*

*****Corresponding author: Lena M. Thorn, email: lena.thorn@helsinki.fi

**Supplemental Table 1.** List of the Finnish Diabetic Nepropathy Study Centers.

| **FinnDiane Study Centers** | **Physicians and nurses** |
| --- | --- |
| **Anjalankoski Health Center** | S.Koivula, T.Uggeldahl |
| **Central Finland Central Hospital, Jyväskylä** | T.Forslund, A.Halonen, A.Koistinen, P.Koskiaho, M.Laukkanen, J.Saltevo, M.Tiihonen |
| **Central Hospital of Åland Islands, Mariehamn** | M.Forsen, H.Granlund, A.-C.Jonsson, B.Nyroos |
| **Central Hospital of Kanta-Häme, Hämeenlinna** | P.Kinnunen, A.Orvola, T.Salonen, A.Vähänen |
| **Central Hospital of Kymenlaakso, Kotka** | R.Paldanius, M.Riihelä, L.Ryysy |
| **Central Hospital of Länsi-Pohja, Kemi** | H.Laukkanen, P.Nyländen, A.Sademies |
| **Central Ostrobothnian Hospital District, Kokkola** | S.Anderson, B.Asplund, U.Byskata, P.Liedes, M.Kuusela, T.Virkkala |
| **City of Espoo Health Center:** |  |
| **Espoonlahti** | A.Nikkola, E.Ritola |
| **Tapiola** | M.Niska, H.Saarinen |
| **Samaria** | E.Oukko-Ruponen, T.Virtanen |
| **Viherlaakso** | A.Lyytinen |
| **City of Helsinki Health Center:** |  |
| **Puistola** | H.Kari, T.Simonen |
| **Suutarila** | A.Kaprio, J.Kärkkäinen, B.Rantaeskola |
| **Töölö** | P.Kääriäinen, J.Haaga, A-L.Pietiläinen |
| **City of Hyvinkää Health Center** | S.Klemetti, T.Nyandoto, E.Rontu, S.Satuli-Autere |
| **City of Vantaa Health Center:** |  |
| **Korso** | R.Toivonen, H.Virtanen |
| **Länsimäki** | R.Ahonen, M.Ivaska-Suomela, A.Jauhiainen |
| **Martinlaakso** | M.Laine, T.Pellonpää, R.Puranen |
| **Myyrmäki** | A.Airas, J.Laakso, K.Rautavaara |
| **Rekola** | M.Erola, E.Jatkola |
| **Tikkurila** | R.Lönnblad, A.Malm, J.Mäkelä, E.Rautamo |
| **Heinola Health Center** | P.Hentunen, J.Lagerstam |
| **Helsinki University Hospital, Department of Medicine, Division of Nephrology** | R.Bergdal, T.Claesson, A.Dufva, N.Elonen, M.Eriksson, J.Fagerudd, M.Feodoroff, D.Gordin, P.-H.Groop, O.Heikkilä, K.Hietala, S.Hägg-Holmberg, F.Jansson Sigfrids, M.Korolainen, J.Kytö, S.Lindh, J.Nicklén, H.Paajanen, K.Pettersson-Fernholm, K.Rimpeläinen, M.Rosengård-Bärlund, M.Rönnback, L.Salovaara, A.Sandelin, M.Saraheimo, S.Satuli-Autere, R.Simonsen, P.Smidtslund, L.Thorn, H.Tikkanen, J.Tuomikangas, A.Tynjälä, K.Uljala, T.Vesisenaho, J.Wadén, A.Ylinen |
| **Herttoniemi Hospital, Helsinki** | V.Sipilä |
| **Hospital of Lounais-Häme, Forssa** | T.Kalliomäki, J.Koskelainen, R.Nikkanen, N.Savolainen, H.Sulonen, E.Valtonen |
| **Hyvinkää Hospital** | L. Norvio, A.Hämäläinen |
| **Iisalmi Hospital** | E.Toivanen |
| **Jokilaakso Hospital, Jämsä** | A.Parta, I.Pirttiniemi |
| **Jorvi Hospital, Helsinki University Central Hospital** | S.Aranko, S.Ervasti, R.Kauppinen-Mäkelin, A.Kuusisto, T.Leppälä, K.Nikkilä, L.Pekkonen |
| **Jyväskylä Health Center, Kyllö** | K.Nuorva, M.Tiihonen |
| **Kainuu Central Hospital, Kajaani** | S.Jokelainen, K.Kananen, M.Karjalainen, P.Kemppainen, A-M.Mankinen, A.Reponen, M.Sankari |
| **Kerava Health Center** | H.Stuckey, P.Suominen |
| **Kirkkonummi Health Center** | A.Lappalainen, M.Liimatainen, J.Santaholma |
| **Kivelä Hospital, Helsinki** | A.Aimolahti, E.Huovinen |
| **Koskela Hospital, Helsinki** | V.Ilkka, M.Lehtimäki |
| **Kotka Health Center** | E.Pälikkö-Kontinen, A.Vanhanen |
| **Kouvola Health Center** | E.Koskinen, T.Siitonen |
| **Kuopio University Hospital** | E.Huttunen, R.Ikäheimo, P.Karhapää, P.Kekäläinen, M.Laakso, T.Lakka, E.Lampainen, L.Moilanen, S. Tanskanen, L.Niskanen, U.Tuovinen, I.Vauhkonen, E.Voutilainen |
| **Kuusamo Health Center** | T.Kääriäinen, E.Isopoussu |
| **Kuusankoski Hospital** | E.Kilkki, I.Koskinen, L.Riihelä |
| **Laakso Hospital, Helsinki** | T.Meriläinen, P.Poukka, R.Savolainen, N.Uhlenius |
| **Lahti City Hospital** | A.Mäkelä, M.Tanner |
| **Lapland Central Hospital, Rovaniemi** | L.Hyvärinen, K.Lampela, S.Pöykkö, T.Rompasaari, S.Severinkangas, T.Tulokas |
| **Lappeenranta Health Center** | P. Erola, L.Härkönen, P.Linkola, T.Pekkanen, I.Pulli, E.Repo |
| **Lohja Hospital** | T.Granlund, K.Hietanen, M.Porrassalmi, M.Saari, T.Salonen, M.Tiikkainen, |
| **Länsi-Uusimaa Hospital, Tammisaari** | I.-M.Jousmaa, J.Rinne |
| **Loimaa Health Center** | A.Mäkelä, P.Eloranta |
| **Malmi Hospital, Helsinki** | H.Lanki, S.Moilanen, M.Tilly-Kiesi |
| **Mikkeli Central Hospital** | A.Gynther, R.Manninen, P.Nironen, M.Salminen, T.Vänttinen |
| **Mänttä Regional Hospital** | I.Pirttiniemi, A-M.Hänninen |
| **North Karelian Hospital, Joensuu** | U-M.Henttula, P.Kekäläinen, M.Pietarinen, A.Rissanen, M.Voutilainen |
| **Nurmijärvi Health Center** | A.Burgos, K.Urtamo |
| **Oulaskangas Hospital, Oulainen** | E.Jokelainen, P-L.Jylkkä, E.Kaarlela, J.Vuolaspuro |
| **Oulu Health Center** | L.Hiltunen, R.Häkkinen, S.Keinänen-Kiukaanniemi |
| **Oulu University Hospital** | R.Ikäheimo |
| **Päijät-Häme Central Hospital** | H.Haapamäki, A.Helanterä, S.Hämäläinen, V.Ilvesmäki, H.Miettinen |
| **Palokka Health Center** | P.Sopanen, L.Welling |
| **Pieksämäki Hospital** | V.Sevtsenko, M.Tamminen |
| **Pietarsaari Hospital** | M-L.Holmbäck, B.Isomaa, L.Sarelin |
| **Pori City Hospital** | P.Ahonen, P.Merisalo, E.Muurinen, K.Sävelä |
| **Porvoo Hospital** | M.Kallio, B.Rask, S.Rämö |
| **Raahe Hospital** | A.Holma, M.Honkala, A.Tuomivaara, R.Vainionpää |
| **Rauma Hospital** | K.Laine, K.Saarinen, T.Salminen |
| **Riihimäki Hospital** | P.Aalto, E.Immonen, L.Juurinen |
| **Salo Hospital** | A.Alanko, J.Lapinleimu, P.Rautio, M.Virtanen |
| **Satakunta Central Hospital, Pori** | M.Asola, M.Juhola, P.Kunelius, M.-L.Lahdenmäki, P.Pääkkönen, M.Rautavirta |
| **Savonlinna Central Hospital** | T.Pulli, P.Sallinen, M.Taskinen, E.Tolvanen, T.Tuominen, H.Valtonen, A.Vartia, S-L.Viitanen |
| **Seinäjoki Central Hospital** | O.Antila, E.Korpi-Hyövälti, T.Latvala, E.Leijala, T.Leikkari, M.Punkari N.Rantamäki, H.Vähävuori |
| **South Karelia Central Hospital, Lappeenranta** | T.Ensala, E.Hussi, R.Härkönen, U.Nyholm, J.Toivanen |
| **Tampere Health Center** | A.Vaden, P.Alarotu, E.Kujansuu, H.Kirkkopelto-Jokinen, M.Helin, S.Gummerus, L.Calonius, T.Niskanen, T.Kaitala, T.Vatanen |
| **Tampere University Hospital** | P. Hannula, I.Ala-Houhala, R.Kannisto, T.Kuningas, P.Lampinen, M.Määttä,H.Oksala, T.Oksanen, A.Putila, H.Saha, K.Salonen, H.Tauriainen, S.Tulokas |
| **Tiirismaa Health Center, Hollola** | T.Kivelä, L.Petlin, L.Savolainen |
| **Turku Health Center** | A.Artukka, I.Hämäläinen, L.Lehtinen, E.Pyysalo, H.Virtamo, M.Viinikkala, M.Vähätalo |
| **Turku University Central Hospital** | K.Breitholz, R.Eskola, K.Metsärinne, U.Pietilä, P.Saarinen, R.Tuominen, S.Äyräpää |
| **Vaajakoski Health Center** | K.Mäkinen, P.Sopanen |
| **Valkeakoski Regional Hospital** | S.Ojanen, E.Valtonen, H.Ylönen, M.Rautiainen, T.Immonen |
| **Vammala Regional Hospital** | I.Isomäki, R.Kroneld, L.Mustaniemi, M.Tapiolinna-Mäkelä |
| **Vasa Central Hospital** | S.Bergkulla, U.Hautamäki, V-A.Myllyniemi, I.Rusk |

**Supplemental Figure 1.** Survival from study baseline stratified by baseline age groups. Between-group comaprisons performed with the Log rank test.


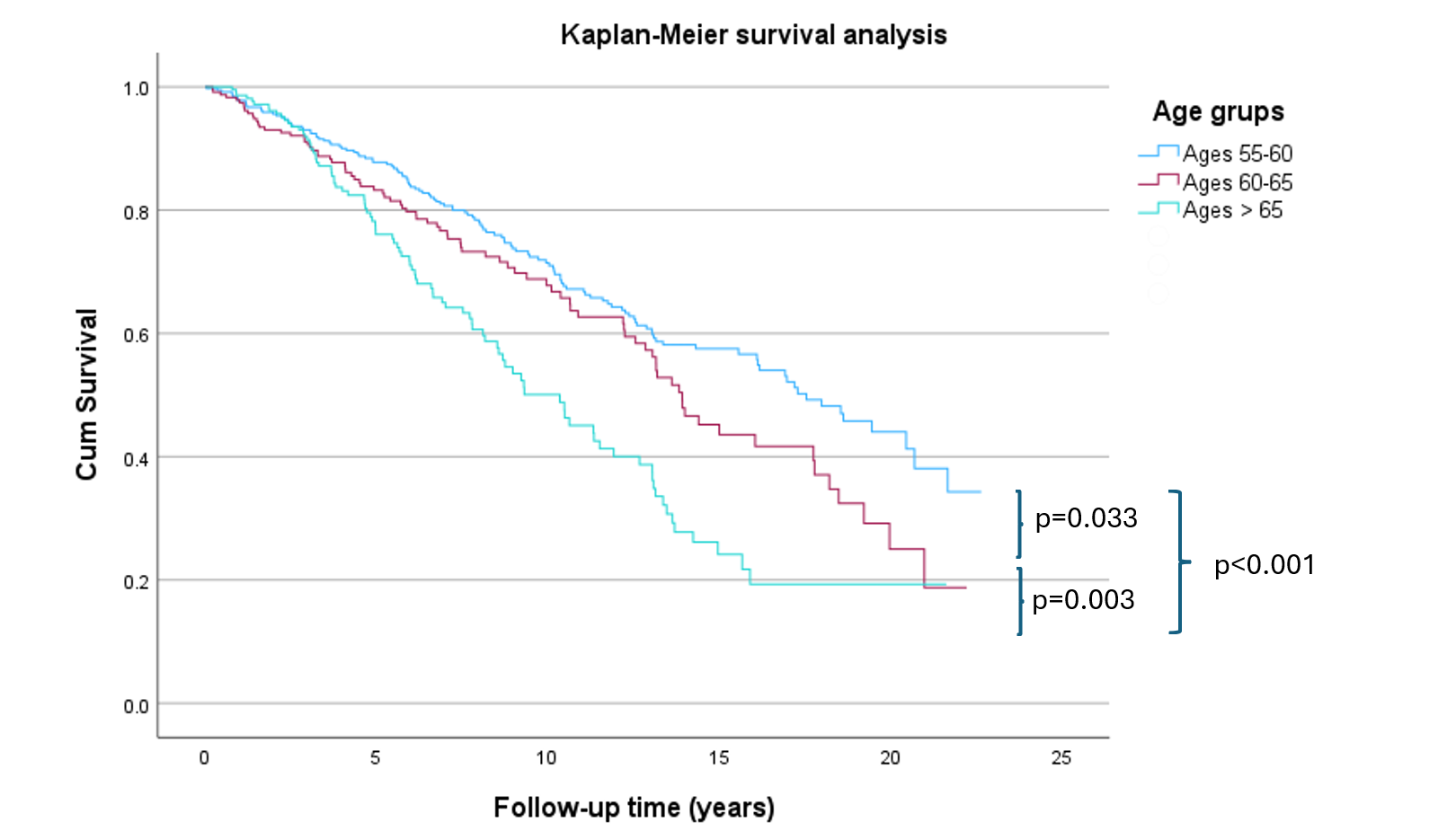

Supplement: Supplementary file 1 — Supplementary Material 1 [file 592_2025_2613_MOESM1_ESM.docx]
